# A survey of well conserved families of C2H2 zinc-finger genes in *Daphnia*

**DOI:** 10.1186/1471-2164-11-276

**Published:** 2010-04-30

**Authors:** Arun Seetharam, Yang Bai, Gary W Stuart

**Affiliations:** 1 Department of Biology, Indiana State University, Terre Haute, IN 47809, USA

## Abstract

**Background:**

A recent comparative genomic analysis tentatively identified roughly 40 orthologous groups of C2H2 Zinc-finger proteins that are well conserved in "bilaterians" (*i.e*. worms, flies, and humans). Here we extend that analysis to include a second arthropod genome from the crustacean, *Daphnia pulex*.

**Results:**

Most of the 40 orthologous groups of C2H2 zinc-finger proteins are represented by just one or two proteins within each of the previously surveyed species. Likewise, *Daphnia *were found to possess a similar number of orthologs for all of these small orthology groups. In contrast, the number of Sp/KLF homologs tends to be greater and to vary between species. Like the corresponding mammalian Sp/KLF proteins, most of the *Drosophila *and *Daphnia *homologs can be placed into one of three sub-groups: Class I-III. *Daphnia *were found to have three Class I proteins that roughly correspond to their *Drosophila *counterparts, dSP1, btd, CG5669, and three Class II proteins that roughly correspond to Luna, CG12029, CG9895. However, *Daphnia *have four additional KLF-Class II proteins that are most similar to the vertebrate KLF1/2/4 proteins, a subset not found in *Drosophila*. Two of these four proteins are encoded by genes linked in tandem. *Daphnia *also have three KLF-Class III members, one more than *Drosophila*. One of these is a likely Bteb2 homolog, while the other two correspond to Cabot and KLF13, a vertebrate homolog of Cabot.

**Conclusion:**

Consistent with their likely roles as fundamental determinants of bilaterian form and function, most of the 40 groups of C2H2 zinc-finger proteins are conserved in kind and number in *Daphnia*. However, the KLF family includes several additional genes that are most similar to genes present in vertebrates but missing in *Drosophila*.

## Background

Zinc-finger proteins (ZFP) represent the largest family of DNA-binding transcription factors in eukaryotes. Although many proteins are predicted to contain single zinc-finger domains, two zinc fingers in close proximity appear to be required for high-affinity DNA binding. There are many diverse subfamilies of zinc-finger proteins in eukaryotes, but the most numerous are the Kruppel-type C2H2 ZFPs. Many of these proteins contain either multiple tandem pairs of zinc-fingers or tandem arrays of three or more zinc-fingers. As transcription factors, they participate generally in the fundamental mechanism of gene expression. However, they usually also play more specific roles in a wide variety of regulated biological processes, including signal transduction, cell growth, differentiation, and development. As part of our collaborative role in annotating the draft genome assembly v1.1 of the *Daphnia pulex *genome[1], we focused our attention on a subset of roughly 40 orthologous groups of C2H2 ZFPs identified in a recent comparative genomic analysis to be well conserved in "bilaterians" (i.e. worms, flies, and humans)[2]. While many of these were known or likely DNA-binding transcription factors encoding proteins with tandem arrays of zinc-fingers (e.g. Zif268, MTF1, TFIIIA, SP1 and KLF), others had only a single zinc finger (e.g. SAP61, SAP62, and Kin17), which generally lack a DNA-binding function [2]. Also included are some genes that encode multiple split pairs of C2H2 zinc-fingers, like Disco

## Results

Previously 39 families of C2H2 ZFP were determined to be present in the common ancestor of bilaterians based on a survey of three organisms: *Homo sapiens*, *Drosophila melanogaster *and *Caenorhabditis elegans*. Although the work described below necessitated the addition of three more conserved families to this collection, two pairs of the original 39 families could also be reasonably combined into single families. Hence, we created a reorganized summary list of 40 orthologous groups of C2H2 ZFP. The resulting list of family members and their accession IDs are provided in Table 1, 2, 3 and 4, while an efficient numerical summary is provided in Table 5. A brief description of the known or proposed function(s) and structural organization for each of these families is provided in series below.

**Table 1:** SP and KLF homologs:

Family	*Homo sapiens*	*Drosophila melanogaster*	*Caenorhabditis elegans*	*Daphnia pulex*	Location (*D. pulex*, v1.1)
SP	SP1 (P08047)	BTD (Q24266)	SPTF1 (NP_001021466)	SP5 (Dappu-114437)	scaffold_130:263756-265967
	SP2 (Q02086)	DSp1 (NP_727360)	SPTF2 (NP_495833)	SP1 (Dappu-315784)	scaffold_15:792601-795915
	SP3/SPR2 (Q02447)	CG5669 (NP_651232)	SPTF3 (NP_493353)	SP8 (Dappu-106303)	scaffold_42:141432-144224
	SP4/SPR1 (Q02446)				
	SP5 (Q6BEB4)				
	SP6/KLF14 (Q3SY56)				
	SP7 (Q8TDD2)				
	SP8 (Q8IXZ3)				

KLF	KLF1 (Q13351)	CG12029 (NP_647822)	KLF1 (NP_497632)	KLF1A (Dappu-48391)	scaffold_16:1551074-1551469
	KLF2 (Q9Y5W3)	CG9895 (NP_611747)	F53F8.1 (NP_507995)	KLF1B (Dappu-51551)	scaffold_26:196741-197325
	KLF3 (P57682)	CG3065 (NP_726393)	MUA1 (AAU20846)	KLF1C (Dappu-243802)	scaffold_26:237754-238940
	KLF4 (O43474)	CABUT (NP_608529)		KLF1D (Dappu-262353)	scaffold_168:128271-129531
	KLF5 (Q13887)	LUNA (NP_995811)		KLF3 (Dappu-27999)	scaffold_1:1214746-1215144
	KLF6 (Q99612)	BTEB2 (NP_572185)		LUNA (Dappu-310992)	scaffold_3:2311937-2324470
	KLF7 (O75840)			CABUT (Dappu-312628)	scaffold_6:1962325-1965508
	KLF8 (O95600)			KLF9 (Dappu-315814)	scaffold_15:985228-986723
	KLF9 (Q13886)			BTEB2 (Dappu-50068)	scaffold_21:551156-551927
	KLF10 (Q13118)			KLF1E (Dappu-262162)	scaffold_164:255538-257421
	KLF11 (O14901)				
	KLF12 (NP_009180)				
	KLF13 (NP_057079)				
	KLF14 (Q8TD94)				
	KLF15 (Q9UIH9)				
	KLF16 (Q9BXK1)				
	KLF17 (Q5JT82)				

The updated list of SP and KLF homologs with their accession numbers found in *Homo sapiens *(Build 36.3 downloaded from ftp://ftp.ncbi.nih.gov/genomes/H_sapiens/ARCHIVE/BUILD.36.3) *Drosophila melanogaster *(Build 4.1, downloaded from ftp://ftp.ncbi.nih.gov/genomes/Drosophila_melanogaster/RELEASE_4.1/), *Caenorhabditis elegans *(Build 7.1, downloaded from ftp://ftp.ncbi.nih.gov/genomes/Caenorhabditis_elegans/ARCHIVE/BUILD.7.1) and *Daphnia pulex *(Version 1.1 downloaded from http://genome.jgi-psf.org/Dappu1/Dappu1.download.ftp.html).

**Table 2:** The updated list of C2H2 zinc finger protein families, that have expansion in *Homo sapiens*, *Drosophila melanogaster*, *Caenorhabditis elegans *and *Daphnia pulex *along with their accession numbers.

Family	*Homo sapiens*	*Drosophila melanogaster*	*Caenorhabditis elegans*	*Daphnia pulex*	Location (*D. pulex*, v1.1)
EGR	EGR1 (NP_001955)	SR (NP_524395)	EGRH1 (NP_510467)	SR (Dappu-96734)	5:1579836-1581770
	EGR2 (NP_000390)		EGRH2 (NP_500019)		
	EGR3 (NP_004421)		EGRH3 (NP_001041062)		
	EGR4 (NP_001956)				

ZFH1/2	ZEB1 (P37275)	ZFH1 (P28166)	ZAG1 (Q94196)	ZFH1 (Dappu-225224)	31:791001-795287
	ZEB2 (O60315)				

ZFH3/4	ZFHX2 (Q9C0A1)	ZFH2 (P28167)	ZC123.3 (O45019)	ZFH2 (Dappu-233159)	2:1855545-1862285
	ZFHX3 (Q15911)				
	ZFHX4 (Q86UP3)				

SPALT	SALL1 (Q9NSC2)	SPALTm (P39770)	SEM4(NP_491997)	SALL (Dappu-111734)	88:97830-103728
	SALL2 (Q9Y467)	SPALTr (NP_523548)			
	SALL3 (Q9BXA9)				
	SALL4 (Q9UJQ4)				

DISCO	BNC1 (Q01954)	DISCO (P23792)	F55C5.11 (Q1ZXU0)	DISCO (Dappu-442650)	91:404649-427757
	BNC2 (Q6ZN30)	DISCO-r (NP_727938)			

GFI	GFI1 (Q99684)	SENS (NP_524818)	PAG3 (O02265)	GPS-A (Dappu-113215)	106:388895-391820
	GFI1b (NP_004179)			GPS-B (Dappu-113216)	106:410992-414351

BLIMP1	PRDM1 (O75626)	BLIMP1 (NP_647982)	BLIMP1 (NP_492723)	PRDM1A (Dappu-319330)	29:157096-160885
	ZNF683 (Q8IZ20)			PRDM1B (Dappu-333601)	247:75181-89061

ZEP	HIV-EP1 (P15822)	SHN (NP_476724)	SMA9 (CAF31491)	SHN (Dappu-226641)	60:513635-519628
	HIV-EP2 (P31629)				
	HIV-EP3 (NP_078779)				

IA1	INSM1 (Q01101)	NERFIN1 (NP_524783)	EGL46 (NP_504694)	NERFIN (Dappu-95880)	3:3754452-3756797
	INSM2 (NP_115983)	NERFIN2 (NP_524300)			

EVI1	PRDM16 (Q9HAZ2)	HAM (Q8I7Z8)	EGL46 (CAA91353)	HAM (Dappu-113201)	106:272281-278224
	EVI1 (Q03112)	CG10348 (NP_609904)			

FEZ	FEZF1(NP_001019784)	CG31670 (NP_608631)	Y38H8A.5 (NP_502594)	FEZL(Dappu-40822)	2:1567364-1568302
	FEZF2 (NP_060478)				

ZFAM1	ZNF706 (Q9Y5V0)	CG18081 (NP_648807)	C01F6.9 (NP_501583)	ZFAM706 (Dappu-230733)	17:391722-392672
		CG15715 (NP_648808)	K10B3.1b (NP_001024783)		

ZFAM2	ZNF342 (Q8WUU4)	CG9650 (NP_727173)	F13H6.1b (NP_001122913)	BCL11 (Dappu-323911)	57:749423-752628
	BCL11A (Q9H165)				
	BCL11B (Q9C0K0)				

ZFAM4	ZNF384 (Q8TF68)	CG2052 (NP_726568)	LIN29 (NP_496545)	RN (Dappu-104384)	30:275315-279360
	ZNF362 (NP_689706)	RN (NP_996178)			
		SQZ (NP_524403)			

ZFAM11/12	KCMF (NP_064507)	CG11984 (NP_731306)	ZK652.6 (NP_001023029)	KCMF (Dappu-310981)	3:2220789-2222830
		CG31642 (NP_723159)			
		CG31835 (NP_723881)			
		CG15286 (NP_609706)			

ZIC	ZIC1 (Q15915)	OPA (P39768)	REF2 (Q94178)	OPA (Dappu-290567)	104:131019-135418
	ZIC2 (O95409)	SUG (Q7K0S9)			
	ZIC3 (O60481)				
	ZIC4(Q8N9L1)				
	ZIC5(Q96T25)				

OVO	OVOL1 (O14753)	OVOrb (P51521)	LIN48 (Q19996)	OVO (Dappu-290491)	191:57-1323
	OVOL2 (Q9BRP0)				

SNAIL	SNAIL3 (NP_840101)	SNAIL (P08044)	K02D7 (NP_499902)	Dappu- 53927	39:954341-955431
	SNAIL2 (O43623)	ESG (P25932)	SCRT1(NP_491001)	Dappu- 129982	110:193847-194734
	SNAIL1 (O95863)	WOR (NP_476601)	CES1 (NP_492338)	ESG (Dappu-347447)	23:1247838-1249532
	hSCRT1 (Q9BWW7)	SCRT (Q24140)		Dappu- 61957	110:238640-239641
	hSCRT2 (Q9NQ03)	CG12605 (NP_995996)			
		CG12391 (NP_610639)			
		CG17181 (NP_612040)			

GLI	GLI1 (P08151)	CI (P19538)	TRA1(NP_001022880)	CI (Dappu-346973)	3:880659-885822
	GLI2 (P10070)	LMD (NP_732811)		LMD (Dappu-118558)	374:17043-18830
	GLI3 (P10071)	SUG (NP_996057)			
	GLIS1 (Q8NBF1)				
	GLIS2 (Q9BZE0)				
	GLIS3 (Q8NEA6)				

ODD Skipped	OSR1 (Q8TAX0)	ODD (P23803)	ODD1 (NP_498552)	Dappu-238529	11:2090652-2095755
	OSR2 (Q8N2R0)	SOB (Q9VQS7)	ODD2 (NP_509032)	Dappu-335367	1:2333632-2339817
		BOWL (Q9VQU9)		BOWL (Dappu-347540)	11:2120184-2122082
				Dappu- 323619	55:200488-243259

**Table 3:** The updated list of C2H2 zinc finger protein families that are resistant to expansion or deletion in *Homo sapiens*, *Drosophila melanogaster*, *Caenorhabditis elegans *and *Daphnia pulex *along with their accession numbers.

Family	*Homo sapiens*	*Drosophila melanogaster*	*Caenorhabditis elegans*	*Daphnia pulex*	Location (*D. pulex*, v1.1)
SAP61	SF3A3 (Q12874)	NOI (O46106)	T13H5.4 (NP_495799)	SF3A3 (Dappu-216576)	scaffold_86:295015-297217

SAP62	SF3A2 (Q15428)	CG10754 (NP_648603)	F11A10.2 (NP_502290)	SF3A2 (Dappu-226064)	scaffold_47:92393-93703

KIN17	KIN17 (O60870)	KIN17 (NP_649212)	Y52B11A.9 (NP_492860)	KIN17 (Dappu-187099)	scaffold_2:3390169-3391601

TF3A	TF3A (Q92664)	TF3A (NP_573161)	TF3A (NP_498067)	TF3A (Dappu-309275)	scaffold_94:449510-451421

ZNF207	ZNF207 (O43670)	CG17912 (NP_609808)	B0035.1 (NP_502124)	ZNF207 (Dappu-225978)	scaffold_45:119210-125698

ZNF277	ZN277 (Q9NRM2)	CG9890 (NP_611750)	ZTF7 (NP_505526)	ZNF277 (Dappu-187894)	scaffold_16:264655-266301

ZFAM5	ZNF622 (Q969S3)	CG6769 (NP_573252)	C16A3.4 (NP_498397)	ZNF622 (Dappu-194021)	scaffold_11:102282-103757

ZFAM6	ZMAT2 (Q96NC0)	CG11586 (NP_647881)	ZK686.4 (NP_498692)	ZMAT2 (Dappu-229015)	scaffold_139:171223-172629

ZFAM7	ZNF598 (Q86UK7)	CG11414 (NP_611932)	C52E12.1 (NP_495439)	ZNF598 (Dappu-323704)	scaffold_56:13693-16737

**Table 4:** The updated list of C2H2 zinc finger families that are absent from one or more organisms along with their accession numbers.

Family	*Homo sapiens*	*Drosophila melanogaster*	*Caenorhabditis elegans*	*Daphnia pulex*	Location (*D. pulex*, v1.1)
YY1	YY1 (P25490)	YY1/PHO (NP_648317.1)		PHO (Dappu-59123)	scaffold_78:192562-193513
	YY2 (O15391)	PHOL (NP_648317)			
	ZFP42 (Q96MM3)				

HNT	RREB1 (Q92766)	PEB (NP_476674)		PEB (Dappu-98615)	scaffold_10:170443-175752

MTF	MTF (Q14872)	MTF (NP_729491)		MTF (Dappu-227205)	scaffold_72:130738-133279

OAZ	EBF (Q2M1K9)	OAZ-PB (NP_001097315)		OAZ (Dappu-95503)	scaffold_3:1704248-1709703
	ZNF521 (NP_056276)				

ZFAM8	JAZF1 (Q86VZ6)	CG12054 (NP_651853)			

ZFAM9	PRDM13 (Q9H4Q3)	CG13296 (NP_648032)		PRDM13 (Dappu-111472)	scaffold_86:123582-125034

CTCF	CTCF (P49711)	CTCF (NP_648109)		CTCF (Dappu-302037)	scaffold_158:218357-221909
	CTCFL (Q8NI51)				

ZXD	ZXDA (P98168)			ZXD (Dappu-54047)	scaffold_39:175871-177445
	ZXDB (P98169)				
	ZXDC (Q2QGD7)				

**Table 5:** Number of C2H2 genes identified in *Daphnia pulex *(Dp) belonging to different families as compared to the updated list of C2H2 zinc finger gene families found in *Homo sapiens *(Hs), *Drosophila melanogaster *(Dm), and *Caenorhabditis elegans *(Ce).

#	*Family*	*Fullname*	*Hs*	*Dm*	*Ce*	*Dp*
1	SP	Specificity protein	8	3	3	3

2	ZIC	Zinc finger protein of the cerebellum/Sugarbabe	5	2	1	1

3	OVO	Protein ovo/Protein shaven baby	2	1	1	1

4	SNAIL	Neural crest transcription factor Slug/Snail/Escargot/Worniu/Scratch	5	7	3	4

5	GLI	Glioma-associated oncogene/cubitus interruptus	6	2	1	2

6	EGR/KROX	Early growth response 1/Transcription factor Zif268/Stripe	4	1	3	1

7	KLF	Kruppel-like zinc finger protein	18	6	3	10

8	ZFH1/2	Zn finger homeobox protein 1/Smad-interacting protein	2	1	1	1

9	ZFH3/4	Zn finger homeodomain protein 3-4	3	1	1	1

10	OSR	odd-skipped-related 2/Sob/Odd/Bowl	2	3	2	4

11	SPALT	Sal-like protein 1/Spalt-like transcription factor	4	2	1	1

12	DISCO	Zinc finger protein basonuclin	2	2	1	1

13	GFI	Growth factor independent protein	2	1	1	2

14	YY1	Yin and yang 1/Delta transcription factor/NF-E1/Pho	3	2	0	1

15	BLIMP	Beta-interferon gene positive regulatory domain I-binding factor	2	1	1	2

16	ZEP	HIV type I enhancer-binding protein 1/Schnurri	3	1	1	1

17	IA1	Insulinoma-associated protein 1/Nerfin	2	2	1	1

18	EVI1	Ecotropic virus integration site 1/Hamlet	2	2	1	1

19	SAP61	Splicing factor 3A subunit 3/Spliceosome-associated protein 61	1	1	1	1

20	SAP62	Splicing factor 3A subunit 2/Spliceosome-associated protein 62	1	1	1	1

21	KIN17	KIN antigenic determinant of recA protein	1	1	1	1

22	HNT	RAS responsive element binding protein 1	1	1	0	1

23	MTF	Metal regulatory element-binding transcription factor 1	1	1	0	1

24	TF3A	Transcription Factor III A	1	1	1	1

25	ZNF207	Zinc finger protein 207	1	1	1	1

26	ZNF277	Zinc finger protein 277	1	1	1	1

27	FEZ	Forebrain embryonic zinc finger protein	2	1	1	1

28	OAZ	Smad- and Olf-interacting zinc finger protein	2	1	0	1

29	ZFAM1	Zinc finger protein 706	1	2	2	1

30	ZFAM2	B-cell lymphoma/leukemia 11A	3	1	1	1

31	ZFAM4	Zinc finger protein 384/Nuclear matrix transcription factor 4	2	3	1	1

32	ZFAM5	Zinc finger protein 622/Zinc finger-like protein 9	1	1	1	1

33	ZFAM6	Zinc finger matrin-type protein 2	1	1	1	1

34	ZFAM7	zinc finger protein 598	1	1	1	1

35	ZFAM8	Juxtaposed with another zinc finger protein; JAZ	1	1	0	0

36	ZFAM9	Zinc finger protein family 9	1	1	0	1

37	ZFAM10	Bromodomain and PHD finger-containing protein	3	1	1	1

38	ZFAM11/12	Potassium channel modulatory factor	1	4	1	1

39	CTCF	CCCTC-binding factor	2	1	0	1

40	ZXD	Zinc finger X-linked Duplicated protein	3	0	1	1

**Figure 1:**
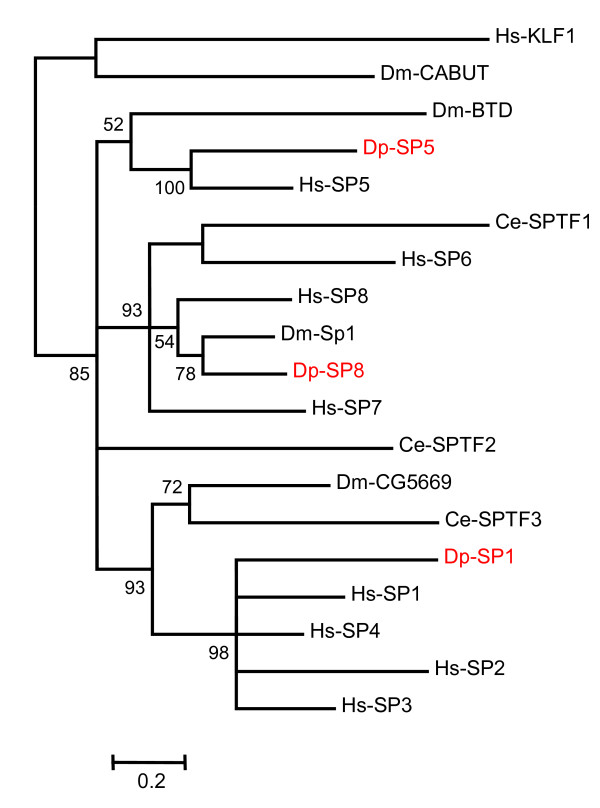
**SP homologs in *Daphnia *correspond to those in *Drosophila***. Bayesian phylogenetic analysis of all SP proteins from Humans, *Drosophila*, *Daphnia *and *C. elegans *rooted with two KLF homologs, Hs-KLF1 and Dm-Cabot. The branch values indicate posterior probability and values greater than 50 are shown (Hs-*Homo sapiens*, Dm-*Drosophila melanogaster*, Ce-*Caenorhabditis elegans *and Dp-*Daphnia pulex*).

**Figure 2:**
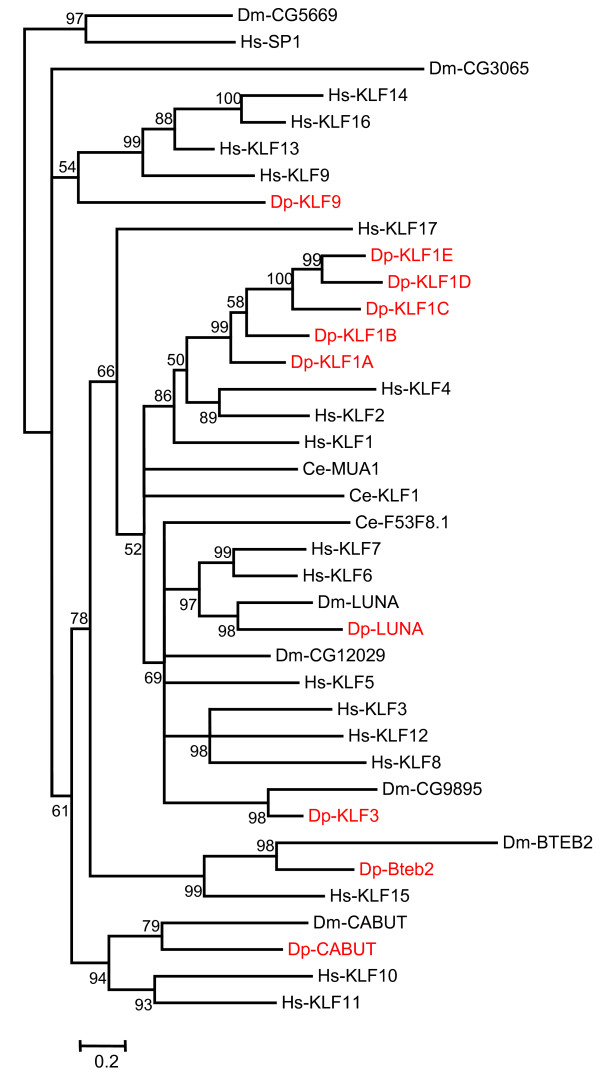
**Additional KLF homologs in *Daphnia *relative to *Drosophila***. Bayesian phylogenetic analysis of all KLF proteins from Humans, *Drosophila*, *Daphnia *and *C. elegans *rooted with two SP homologs, Hs-SP1 and Dm-CG5669. The branch values indicate posterior probability and values greater than 50 are shown (Hs-*Homo sapiens*, Dm-*Drosophila melanogaster*, Ce-*Caenorhabditis elegans *and Dp-*Daphnia pulex*).

**Figure 3:**
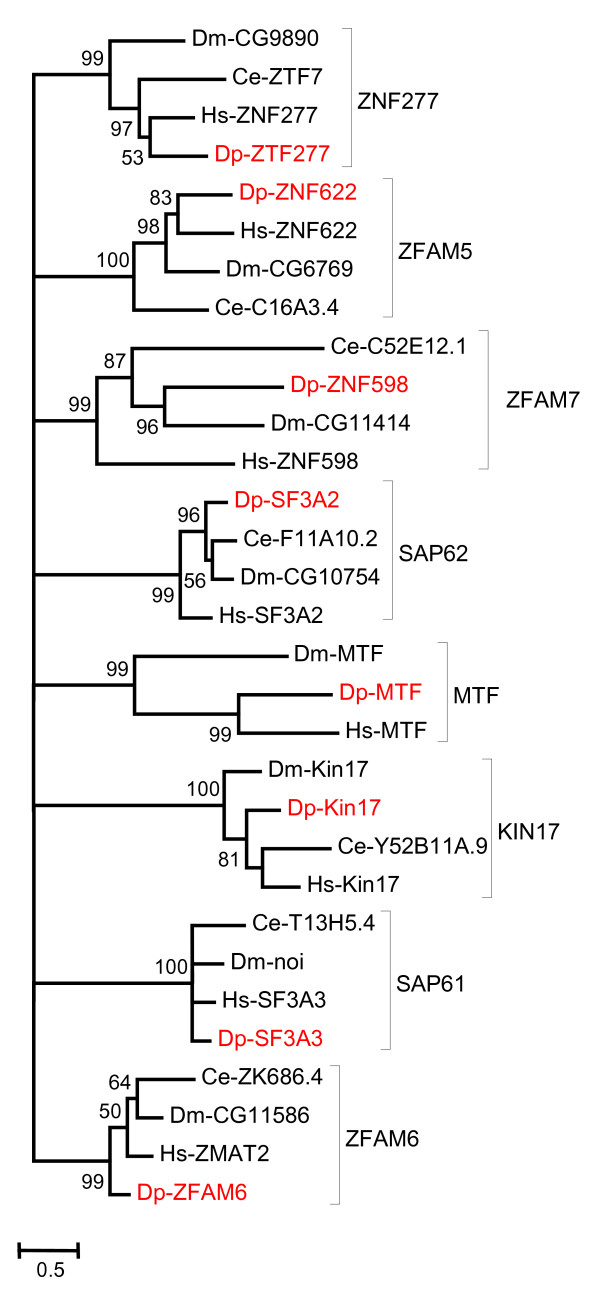
**Bayesian phylogenetic analysis of C2H2 ZNF families that appear to be resistant to deletion/expansion in bilaterians (other than MTF)**: Proteins of ZNF277, Zfam5, Zfam7, SAP62, KIN17, SAP61 and Zfam6 families that have one member in each family and MTF family that has missing member in *C. elegans*, were used to construct phylogenetic tree. The branch values indicate posterior probability and values greater than 50 are shown (Hs-*Homo sapiens*, Dm-*Drosophila melanogaster*, Ce-*Caenorhabditis elegans *and Dp-*Daphnia pulex*).

**Figure 4:**
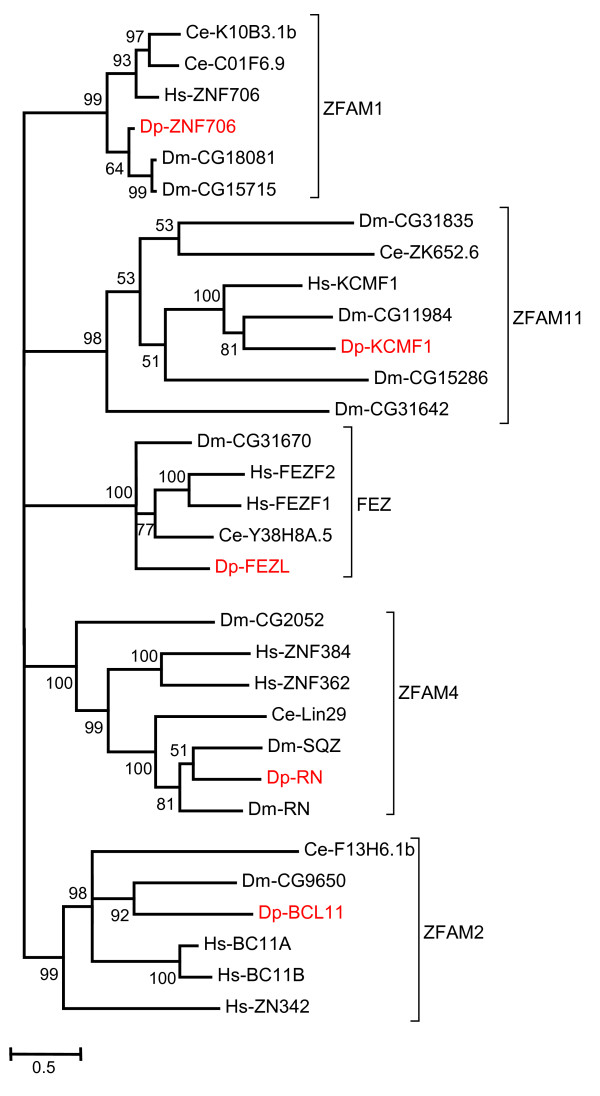
**Bayesian phylogenetic analysis of C2H2 ZNF families with expansions in organisms other than *Daphnia***. Proteins of Zfam1, Zfam11, Fez, Zfam4 and Zfam2 family all having one member in *Daphnia *but more than one member in other genomes. The branch values indicate posterior probability and values greater than 50 are shown (Hs-*Homo sapiens*, Dm-*Drosophila melanogaster*, Ce-*Caenorhabditis elegans *and Dp-*Daphnia pulex*).

**Figure 5:**
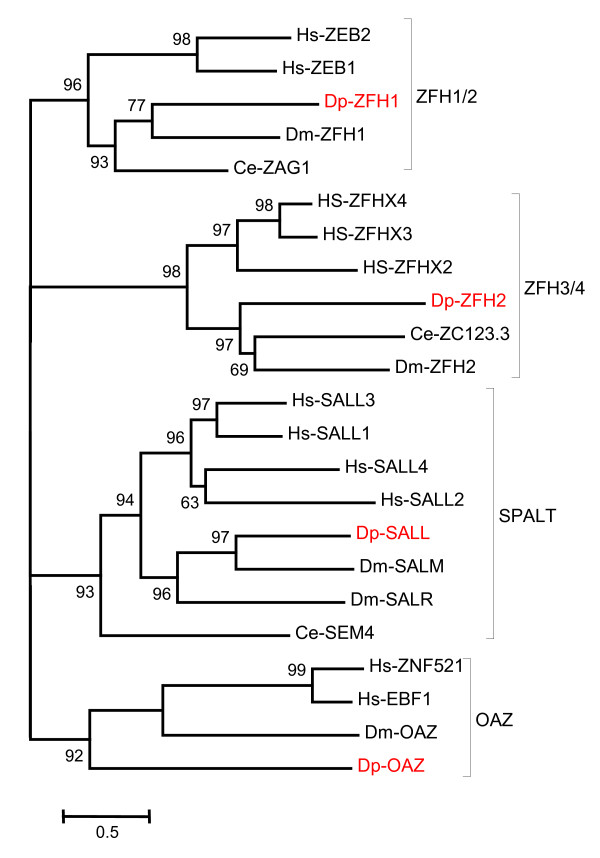
**Bayesian phylogenetic analysis of C2H2 ZNF families with expansions in organisms other than *Daphnia***. Families ZFH1/2, ZFH3/4 and OAZ have additional homologs only for Humans, Spalt family has additional homologs for both Humans and *Drosophila *and all families have one homolog for the *Daphnia *genome. Oaz family has no homolog for *C. elegans*. The branch values indicate posterior probability and values greater than 50 are shown (Hs-*Homo sapiens*, Dm-*Drosophila melanogaster*, Ce-*Caenorhabditis elegans *and Dp-*Daphnia pulex*).

**Figure 6:**
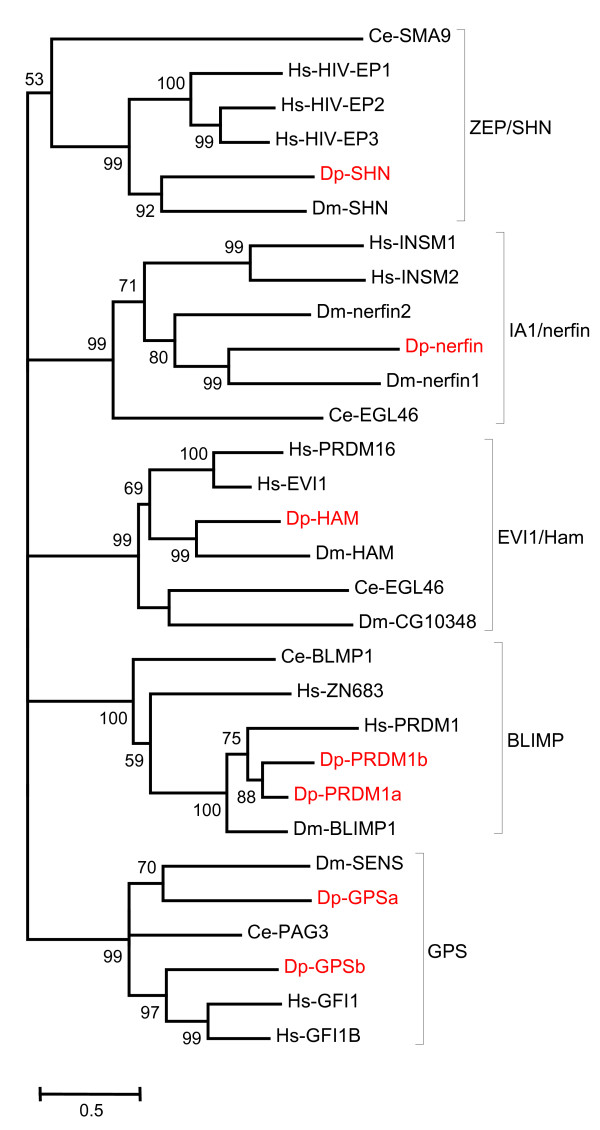
**Bayesian phylogenetic analysis of C2H2 ZNF familes with expansions in bilaterians**. Family Zep/Shn has additional homologs only in Humans, families IA1/Nerfin and Evi1/Ham have additional homologs in Humans and *Drosophila *and Families Blimp and GPS have additional homologs in Humans and *Daphnia *but not in *Drosophila*. The branch values indicate posterior probability and values greater than 50 are shown (Hs-*Homo sapiens*, Dm-*Drosophila melanogaster*, Ce-*Caenorhabditis elegans *and Dp-*Daphnia pulex*).

**Figure 7:**
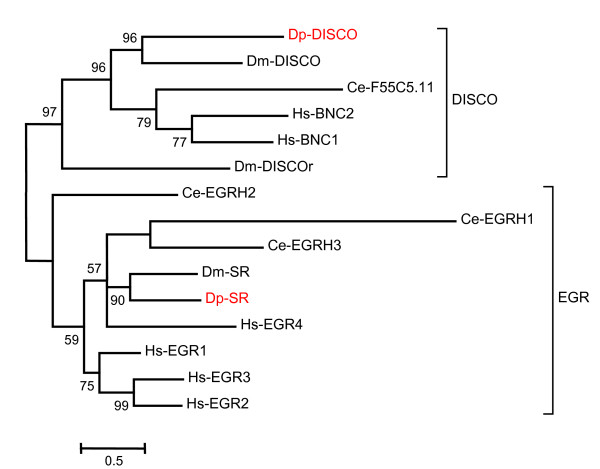
**Bayesian phylogenetic analysis of C2H2 ZNF families Disco and EGR**. Family Disco has additional homologs in humans and *Drosophila *and EGR has additional homologs in Humans and *C. elegans*. The branch values indicate posterior probability and values greater than 50 are shown (Hs-*Homo sapiens*, Dm-*Drosophila melanogaster*, Ce-*Caenorhabditis elegans *and Dp-*Daphnia pulex*).

**Figure 8:**
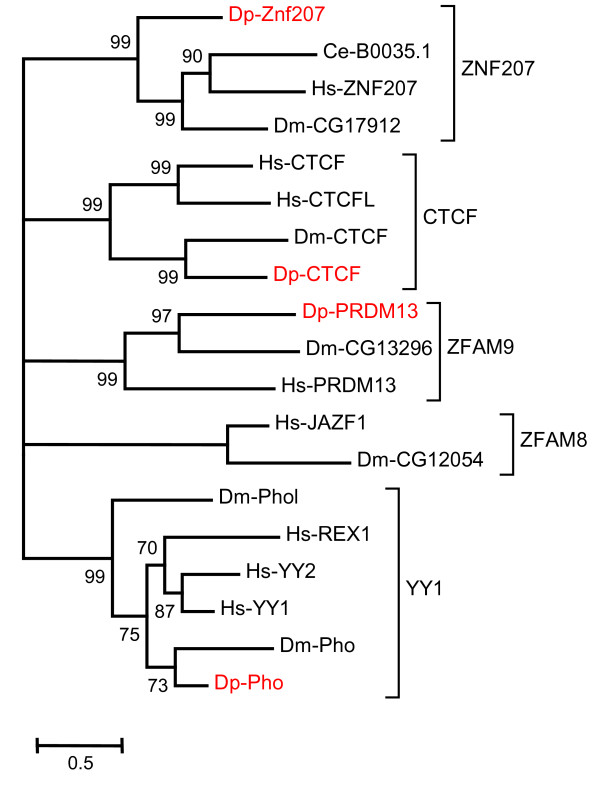
**C2H2 ZNF absent from one or more organisms**. Except for the family ZNF207 all other families in this tree is missing homolog in at least one genome. Families CTCF, Zfam9 and YY1 have a missing member for *C. elegans *and family Zfam8 is missing homolog in both *Daphnia *and *C. elegans*. The branch values indicate posterior probability and values greater than 50 are shown (Hs-*Homo sapiens*, Dm-*Drosophila melanogaster*, Ce-*Caenorhabditis elegans *and Dp-*Daphnia pulex*).

A relatively rigorous assessment of homology/orthology is provided by a set of carefully constructed phylogenetic trees (see Figure 1, 2, 3, 4, 5, 6, 7 and 8 and supplementary Additional file 1 and 2). These trees also serve to summarize various evolutionary events of interest, such as presumptive gene losses, duplications, and lineage-specific expansions. Larger C2H2 Zinc-finger families (i.e. Sp and KLF) are presented within separate trees (Figures 1 and 2), while most of the remaining families are displayed in a short series of multi-family summary trees (Figure 3, 4, 5, 6, 7 and 8). The latter provide additional evidence that member gene clusters represent distinct families of correctly identified homologs and putative orthologs. Evidence for the expression of almost all of these genes at the RNA level was obtained using a NimbleGen tiling array [3-5] and the EST data available at JGI portal [1,6]. Only three genes lack any evidence of expression to date (KLF2D, ZFam9, and FEZL).

### Sp/KLF, the Largest Family of C2H2 ZFP (Figures 1 &2)

The Sp/KLF proteins are DNA-binding transcription factors each containing 3 zinc-fingers. Although Sp and KLF factors are closely related, they occupy distinct branches on a combined evolutionary tree. To facilitate presentation, however, it was most convenient to separate these families into two distinct trees, each rooted with two members from the other family (Figures 1 and 2). Although frequently described as simple transcription factors (especially Sp), many are known to interact with particular sets of chromatin remodeling complexes to facilitate transcriptional activation or repression [7]. In vertebrates, Sp/KLF proteins are grouped into three classes that tend to correlate with the type of chromatin remodeling complexes they utilize. Conveniently, most of the invertebrate proteins can also be assigned to one of these classes [8]. dSP1, btd, and CG5669 correspond to three distinct subsets of Sp-Class I proteins Sp7/8, Sp5, and Sp1/2/3/4, respectively (Figure 1). Luna, CG12029, and CG9895 correspond to the KLF-Class II proteins KLF6/7, KLF5, and KLF3/8/12, respectively (Figure 2). Bteb2 and Cabot correspond to KLF-Class III proteins KLF15 and KLF9/10/11/13/14/16, respectively (Figure 2). The two remaining fly proteins (CG3065 and hkb) are difficult to place unambiguously. The former shows roughly equal similarity to members of both the Sp and KLF subfamilies, while the latter appears to be a highly diverged and relatively unique member of the Sp/KLF family. Information concerning the function of many of these proteins is available in a variety of organisms [9,10]. In vertebrates, some Sp/KLF proteins produce early embryonic lethals when mutated (Sp1, KLF5), some are known to affect behavior and/or the development of structures within the brain (Sp4, Sp8, KLF9), and others affect development of the blood cells (Sp3, KLF1, KLF3), goblet cells in the colon, or bone cells (Sp7). In *Drosophila*, buttonhead (btd) is important for the development of head structures, and both btd and dSp1 affect development of the mechano-sensory organs [11]. Cabot (cbt) also affects sensory organ development, as well as dorsal closure [12]. Perturbations of luna expression via RNA interference or over-expression during early *Drosophila *embryogenesis leads to developmental arrest at different embryonic stages [13]. *Daphnia *appear to contain three Sp homologs (Sp8, Sp5, Sp4), each of which correspond to specific counterparts (SP1, btd, CG5669) in *Drosophila*. In contrast, the KLF gene family in *Daphnia *includes 10 distinct genes, 4 more than the complement in *Drosophila*. Dp-KLF3, Dp-Luna, Dp-Cabot, and Dp-Bteb2 appear to correspond to the fly genes CG9895, Luna, Cabot, and Bteb2, respectively. No clear homologs of KLF17 or CG12029 are apparent in *Daphnia*. In contrast, six KLF genes in *Daphnia *with no direct homologs in *Drosophila *seem to correspond to two subfamilies of KLF found in the vertebrate genome. Five of these, Dp-KLF1A through Dp-KLF1E, may represent a species specific gene expansion that roughly corresponds to an independent expansion in humans that includes KLF1/2/4. The sixth may be a lone homolog of the vertebrate expansion that includes KLF 9/13/14/16.

### C2H2 ZFP resistant to deletion/expansion

Some C2H2 ZFP exist as single copy family members in all 4 genomes. Hence, these genes appear to be relatively resistant to deletion or expansion over evolutionary time. ZNF277 is one of several examples (Figure 3). This gene encodes a protein with five C2H2 zinc fingers. The function of this gene is not well understood. In humans, this gene is expressed in early embryonic tissues, parathyroid adenoma, and chronic lymphocytic leukemia suggesting that this gene might be involved in differentiation [14].

ZFAM5 is also known as ZNF622 or Zinc finger related protein 9 (ZRP9) and is highly conserved almost universally in eukaryotes. Most homologs have 4 zinc-fingers (Figure 3). ZFAM5 was originally identified in mouse as a cellular MPK38 serine/threonine kinase binding protein that may be involved in early T cell activation and embryonic development [15]. In humans this gene is responsible for interaction with the ubiquitously expressed MYB-B transcriptional regulator. The role of this gene in other organisms is not well understood [16].

ZFAM6 is also known as Zinc finger Matrin Type 2 (ZMAT2). It is a highly conserved gene present in most eukaryotes. Little is known about its function. A single homolog of this gene was also found in *Daphnia *(Figure 3). All family members possess one U1-like zinc finger. This type of C2H2 zinc finger is also present in the protein matrin, the U1 small nuclear ribonucleoprotein C, and other RNA-binding proteins.

ZFAM7 is also known as Zinc Finger 598 (ZNF598) in vertebrates. This gene is highly conserved in most eukaryotes and is present as a single homolog in most species, including *Daphnia *(Figure 3). The gene has five C2H2 zinc fingers. The function of this gene is largely unknown.

SAP61 (Splicosome Associated Protein 61) is also known as Splicosome factor 3a subunit 3 (SF3a3), while SAP62 (Splicosome Associated Protein 62) is also known as Splicosome factor 3a subunit 2 (SF3a2). These sequences do not cluster within a phylogenetic tree of zinc finger genes (Figure 3). However, since they share common function by virtue of being subunits of the same protein complex involved in RNA splicing, we described them together. A single homolog for these genes is present in almost all species of eukaryotes, including *Daphnia*. Both have a highly conserved U1-like zinc finger that is typical for RNA binding proteins. These are essential proteins, required for the formation of SF3a and functional U2 snRNP. Together with SF3b, SF3a binds to the 12S U2 snRNP, which contains a common core of seven Sm proteins and the U2-specific proteins U2-A and U2-B [17,18].

KIN17 is present in almost all eukaryotic organisms. *Daphnia *have a single homolog for this gene. KIN17 includes a single highly conserved U1-like zinc finger and is likely to be involved in cellular response to DNA damage, gene expression, and DNA replication. The KIN17 protein shares sequence homology with bacterial RecA protein over 40 residues near the c terminus. KIN 17 is ubiquitously expressed in mammals at low levels, but is up regulated after exposure to UV and ionizing radiation. KIN17 binds to DNA targets found in hot spots of illegitimate recombination [19-21].

A single homolog of ZNF207 is present in almost all vertebrates and invertebrates. *Daphnia *also has a single homolog (Figure 8). The N terminus of this protein contains 2 C2H2 type zinc fingers. This gene is expressed ubiquitously in humans [22], but the exact function is still not clear.

### C2H2 ZFP with expansions in organisms other than *Daphnia*

The *Daphnia *genome appears to deploy a relatively efficient set of well conserved C2H2 ZFP because many C2H2 subfamilies have undergone lineage specific expansions in other genomes. In fact, 12 of the 40 well conserved families considered here show expansions in flies or humans, but not in *Daphnia*. For instance, Zfam1 (Figure 4) codes for a small peptide of 70 to 80 residues that contains one C2H2 type zinc finger. This gene is conserved in chordates and insects. Lineage specific independent duplications have generated 2 homologs in *Drosophila *and 2 in *C. elegans*. *Daphnia *have just one homolog for this gene that clusters with the *Drosophila *homolog. The function of these genes is not well understood.

Humans have three ZFAM2/BCL11 homologs: Bcl11A, Bcl11B, and ZNF342/Zfp296. BCL11A has 5 zinc-fingers, and is a homolog of the murine gene Evi9. Evi9 was found to be deregulated in mouse myeloid leukemias induced by proviral integration. Hence Evi9 has characteristics of a dominant oncogene. Human EVI9/BCL11A is expressed in CD341 myeloid precursors. BCL11A is known to be involved in both Hodgkins and non-Hodgkins B-cell lymphoma [23,24]. BCL11A acts as a proto-oncogene for B-cell lymphoma, as a recessive oncogene for T-cell lymphoma, and is apparently required for the expression of some globin genes [25]. Bcl11B also appears to act like a recessive oncogene for T-cells [26]. The single *Daphnia *homolog appears closely related to the *Drosophila *version (Figure 4). Apparently, duplication in mammals led to 2 or three versions of this gene, two of which became key regulators in hematopoetic lineages, while the third appears to function in the nervous system. ZNF342 has been indirectly implicated in the suppression of gliomas.

ZFAM4 is also known as Rotund and Squeeze in *Drosophila*, and Lin-29 (abnormal cell LINeage family member 29) in C. elegans. There appears to be 3 homologs for this family in humans and one additional homolog in *Drosophila*. Most genes in this family have 5 zinc fingers while two human genes (ZNF384 and ZNF362) and one *Drosophila *gene (CG2052) have an additional zinc finger. The *Daphnia *homolog clusters with *Drosophila *Rotund and squeeze (Figure 4). Roughened eye (Roe) is a part of the rotund gene represented by a different transcript by using a different promoter. They both share the C-terminal region and zinc finger domain but differ in their N-terminal regions. Roe appears to have a role in eye development in the embryos [27]. The *C. elegans *gene *lin-29 *is required for terminal differentiation of the lateral hypodermal seam cells during the larval-to-adult molt and proper vulva morphogenesis. CIZ (CAS interacting Protein or ZNF384) is a nucleocytoplasmic shuttling protein that binds to CAS elements found in promoters of matrix metalloproteinases (MMPs) genes that produce enzymes used to degrade the extracellular matrix proteins [28].

ZFAM11 is also known as KCMF (Potassium channel modulatory factor). It is present in most vertebrates and invertebrates. *Daphnia*, like most vertebrates, has a single copy. *C. elegans *too have a single member for this family which suggests that the gene has been duplicated specifically in flies/insects (Figure 4). All of these genes have a highly conserved region which includes one ZZ type zinc finger and one C2H2 type zinc finger. The ZZ motif is known to bind two zinc ions and most likely participates in ligand binding or molecular scaffolding. In vertebrates, KCMF1 is shown to have intrinsic E3 ubiquitin ligase activity. Studies indicated that KCMF1 is involved in regulating growth modulators [29]. The function of KCMF1 homologs in *Drosophila *and worms is poorly understood.

Fez typically has 6 zinc-fingers, and is a likely ortholog of the human genes ZNF-312 and 312-like. The forebrain expression pattern for this gene was first described in zebrafish, where there is also a second homolog known as Fezl. Fez expression is first detected in the anterior presumptive neuroectoderm of zebrafish during epiboly. Expression becomes focused in the rostral forebrain region during somitogenesis. By 24 hrs, expression is largely restricted to the telencephalon and anterior/ventral region of the diencephalon. Hence Fez is an early marker of anterior neuroectoderm and appears to regulate forebrain development [30]. In mammals, these proteins appear to regulate olfactory-bulb development and neuronal differentiation in the cortex [31,32]. Double knockouts indicate that together FEZ and FEZL play a role in rostral brain patterning in mouse. *Drosophila *and *Daphnia *appear to have only one homolog of Fez (Figure 4). There is little information about the role of the Fez protein in these organisms.

The zinc-finger E-box binding (ZEB) homeobox genes (Figure 5) were previously described as two separate families in chordates (ZEB1/ZFHX1A/ZFH1 and ZEB2/ZFHX1B/ZFH2) or as the zinc finger axon guidance gene (ZAG1) in *C. elegans*. The gene is conserved in most bilaterians and usually has a homeodomain flanked by two separate, highly conserved zinc finger clusters. Most have 6 C2H2 type zinc fingers present as triplets distributed over the length of the gene. The E-box-like target sites overlap with those bound by the Snail family of zinc-finger proteins. ZEB proteins can repress target genes transcription by recruiting the CtBP (C-terminal-binding protein) co-repressor, which is a component of the larger repressor complex containing HDAC (histone deacetylase) and PcG (polycomb group proteins) [33]. ZEB1 and ZEB2 in humans are expressed in several tissues including muscle and CNS. They are also expressed in T lymphocytes and during skeletal differentiation. They are mediators of epithelial dedifferentiation in mammals through the down-regulation of E-cadherin expression [34]. ZEB2, also known as Smad Interacting Protein 1 (SIP1), is over expressed in cancer cells, causing loss of cell polarity and facilitating migratory and invasive behavior. SIP1 is also involved in the development of the neural-crest, the central nervous system, the septum of the heart, and establishment of the midline [35]. Mutations in SIP1 cause Mowat-Wilson Syndrome, a mental retardation syndrome in humans [36,37]. In *Drosophila*, ZFH1 is a transcriptional repressor that regulates differentiation of muscle and gonadal cells, but is also expressed in the CNS [38]. ZAG-1 in *C. elegans *also acts as a repressor that regulates multiple, discrete neuron-specific aspects of terminal differentiation, including cell migration, axonal development, and gene expression [39]. *Daphnia *have a single homolog.

ZFHX genes encode zinc-finger homeobox containing proteins previously described as two separate families (ZFH3 and ZFH4) in bilaterians. In vertebrates this gene appears to have undergone duplications generating 2 or more additional homologs (Figure 5). In humans, there are 3 homologs (ZFHX2, ZFHX3 and ZFHX4). Family members usually contain 8 or more C2H2 zinc fingers distributed throughout the gene. The *Daphnia *homolog has 11 C2H2 type zinc fingers. ZFHX genes are thought to be important regulators of neuronal differentiation [40]. Like most homeotic genes, these genes are also involved in embryonic morphogenesis. ZFHX3, also known as AT motif binding factor 1 (ATBF1), inhibits cell growth and differentiation and may play a role in malignant transformation. It has been shown that it is potential tumor suppressor genes that repress alpha-fetoprotein (AFP) whose altered expression may lead to development of carcinoma in various tissues [41]. ZFHX4 expression is important for neuronal and muscle differentiation, and in rats it is shown to be involved in neural cell maturation [42]. The *Drosophila *homolog ZFH2 is involved in establishing proximal-distal domains in the developing wing disc [43].

Spalt-like (SALL) proteins have a variable number of zinc-fingers: the worm homolog has 6, flies and *Daphnia *have 7, and chicken/human have 7 or 9, depending on the homolog. The two fly genes, Spalt-major (SALM) and Spalt-related (SALR), appear to have duplicated independently from the ancestral gene that also gave rise to the four human homologs (Figure 5). SAL in flies is required for proper development of the trachea, for vein patterning in wing imaginal discs, and for bristle formation in the thorax. In the later case, SAL acts through regulation of pro-neural gene expression [44]. Nervous system expression is a well-conserved aspect of SAL gene function from worms to man. Mutations in the worm homolog (SEM4) affect development of neurons and sensory organs, while mutations in a human homolog (SALL1) result in sensorineural hearing loss and mental retardation (but also anal, genital, and limb malformation). Flies lacking both SALM and SALR are also deaf, with limb and genital malformations potentially analogous to those in humans [45].

ZEP homologs (Figure 6) are referred to as Schnurri (Shn) in flies and SMA9 in worms. There are three or more homologs in vertebrates, but just one in worms, flies, and *Daphnia*, suggesting a lineage specific expansion exclusive to vertebrates. ZEP proteins generally have five C2H2 zinc-fingers divided into two pairs and a solitary medial finger (missing in some homologs). The worm homologs have an additional C-terminal zinc finger pair with little similarity to the other family members, implying a unique function. The C terminus of this protein is also unique to worm [46]. ZEP/Shn/SMA9 homologs are involved in BMP signaling. Bone morphogenetic proteins (BMPs) are members of the transforming growth factor β (TGFβ) family that regulate various biological process including embryonic axes, cell fate determination, proliferation and apoptosis in both invertebrate and vertebrate model systems. In mouse, Shn-2 is required for efficient transcription of *PPARγ2*, which in turn drives the expression of several genes involved in adipocyte differentiation [47]. Shn3 in mouse is a transcriptional regulator of Runx2, which in turn activates several osteoblast differentiation genes. In humans Shn3 is involved in T-cell proliferation, cytokine production, effector function, and inflammatory response [48]. In worms, SMA9/SMAD affects body size regulation and male tail patterning in worms [49]. In *Drosophila*, Shn binds to SMAD to form the repression complex controlling brinker (Brk), which is a transcriptional repressor of the Dpp gene. Dpp is involved in anterior-posterior patterning and cell proliferation in the wing blade [50].

The Insulinoma Associated gene (IA1) of vertebrates is called Nerfin in flies and Egg laying 46 (EGL46) in worms. *Daphnia *and C. elegans appear to have just one homolog with two conserved zinc-fingers. This gene appears to have undergone independent duplications in the human and fly lineages, giving rise to two paralogs in each (Figure 6). IA homologs are involved in various aspects of neuronal differentiation including cell fate specification, axon guidance decisions and cell migration. In Humans IA1 promotes pancreatic and intestinal endocrine cells development [51]. Recent reports for mice and zebra fish imply that its role in neurogenesis is conserved across vertebrates as well as invertebrates [52,53].

Hamlet is also called PR domain zinc finger protein 16 (PRDM16) or ecotropic virus integration site 1 (EVI-1) homolog in vertebrates, Hamlet in *Drosophila *and Egg laying 43 (EGL43) in *C. elegans*. *Daphnia *has one homolog. Independent duplications in insects and vertebrates appear to have generated two paralogs each in their respective clades (Figure 6). All homologs contain an N-terminal PR (PRD1-BF1-RIZ1) homology domain followed by a group of six zinc fingers and a group of three additional ZFs at the C-terminus. In *Drosophila*, hamlet functions as a binary genetic switch specifically affecting the dendritic branching structure of external sensory (ES) neurons in the peripheral nervous system [54]. In C. elegans, egl-43 encodes two transcription factors that act to control HSN migration and phasmid neuron development, presumably by regulating other genes that function directly in these processes [55]. The murine homolog Evi-1 was obtained from a common site of viral integration in murine myeloid leukemia. The human homolog MDS1/EVI1 is transcriptionally activated by several recurrent chromosomal aberrations like acute myeloid leukemia (AML) and myelodysplastic syndrome (MDS). Recently, another homolog of HAM called *MEL1 *(*MDS1/EVI1*-like gene 1) was identified as a member of the *EVI1 *gene family and also as a PR domain member (PRDM16), all of which are implicated in neural development [56]. The disruption of the PR domain of this gene can cause leukemia. A partial disruption of the *Mds1/Evi1 *locus in mouse leads to multiple defects causing mid-gestation lethality, including defects of hypocellularity in the neuroectoderm and a failure of peripheral nerve formation [57].

The stripe gene (Sr) in *Drosophila *functions in the epidermis to facilitate cellular recognition of myotubules (Figure 7). Hence, stripe mutants exhibit a disruption in myotubule patterning. Stripe is a member of the EGR (early growth response) family of transcription factors. The Egr transcription factors are rapidly induced by diverse extracellular physiological/chemical stimuli within the vertebrate nervous system. These proteins possess 3 zinc fingers. Another member of this family, Krox20, is known to be involved in development of the hindbrain and neural crest in mammals. Analysis of mouse knockouts has demonstrated that Egr2/Krox-20 is important for hindbrain segmentation and development, peripheral nervous system (PNS) myelination, and Schwann cell differentiation [58]. Krox20 expression correlates with the onset of myelination in the PNS. Egr-1 and egr-3 are also both implicated in learning and memory [59]. EGR-1 was shown to be induced in specific subregions of the brain during retrieval of fear memories. Knockout mice further showed that egr-1 was essential for the transition from short- to long-term plasticity and for the formation of long-term memories. In T-cells of the immune system, egr-3 and egr-4 work together with NF-kappaB to control transcription of genes encoding inflammatory cytokines. Egr-2 and egr-3 can also inhibit T cell activation [60]. The egr genes are distantly related to the Wilm's tumor (WT) gene. The latter, like the distantly related klumpfuss gene in *Drosophila*, has 4 zinc fingers rather than 3. The single *Daphnia *homolog is seen to be most similar to that in *Drosophila *(Figure 3).

Disco homologs have 4 to 6 zinc fingers in a paired arrangement. In humans, Basonuclin 1 and 2 (BNC1 & 2) correspond to the Disco and Disco-r genes present in flies (Figure 7). In humans basonuclin is expressed in keratinocytes, germ cells, cornea, and lens epithelia. BNC2 mRNA is abundant in cell types that possess BNC1 but is also found in tissues that lack BCN1, such as kidney, intestine, and uterus [61]. In keratinocytes, BNC maintains proliferative capacity and prevents their terminal differentiation [62]. Recently, it has been suggested that BNC2 is also involved in mRNA export, nonsense-mediated decay, and/or polyadenylation [61]. Both BNC's activate the expression of rRNA genes. Disconnected *(Disco) *and disco-related *(Disco-r) *are two functionally redundant, neighboring genes localized on the fly X chromosome that may act in combination with the homeotic genes *deformed (dfd) *and Sex Combs Reduced *(SCR) *to specify gnathal structures in *Drosophila *[63,64]. The ancestral Disco gene appears to have undergone independent duplication events in the human and fly lineages. *Daphnia *appear to have just one homolog.

### C2H2 ZNF homologs duplicated in *Daphnia *but not *Drosophila*

Lineage Specific duplications of well conserved C2H2 ZFP in *Daphnia *appear to be rare. GPS homologs (GFI/PAG/Sens) generally have 6 tandem zinc-fingers (Figure 6). The C elegans homolog (PAG3) has only 5 fingers (missing the first one). The *Drosophila *homolog (SENS) has only 4 fingers (missing the first two). The two *Daphnia *homologs cluster together and thus appear to be a recent duplication independent of that producing the two GFI homologs in humans. One *Daphnia *homolog (GPSa) has a unique 5aa insertion between fingers 1 and 2. GPS proteins are involved in hematopoesis and neurogenesis. The hematopoetic functions of vertebrate GFI-1 and GFI-1B (6 fingers) appear to be exchangeable, but distinct, due to differential cell-type specific expression. They differ in their ability to facilitate late maturation of inner ear neurons [65]. While generally known as transcriptional repressors, some act as transcriptional activators and/or conditional repressors (like Sens). The single worm homolog serves to repress touch neuron gene expression in interneuron cells [66]. The *Drosophila *Senseless gene is required for normal sensory organ development [67]. The two *Daphnia *GPS genes are closely linked on the same scaffold. A domain required for transcriptional repression, the SNAG domain, is found only in vertebrate GFI proteins to date; hence no SNAG domain is seen in the *Daphnia *homologs [68].

PRDM/Blimp proteins are Putative Positive Regulatory Domain/B-lymphocyte induced maturation proteins. These proteins have 5 fingers, but the 5th finger is relatively poorly conserved and has a C2HC structure. A single PRDM1/Blimp gene is found in humans, one in flies, and one in worms (Figure 6). Blimp1 expression in the tracheal system of *Drosophila *embryos was found to be important for the development of this tissue. Blimp1 is also induced by ecdysone, and reduced Blimp1 expression results in prepupal lethality [69]. Blimp1 is expressed in many other tissues of *Drosophila*. Blimp is similarly expressed in many different tissues in vertebrates, where it is known to play important roles in embryogenesis, germ cell determination, specification in nerve and muscle cells, linage determination in epidermis, and B-cell maturation [70-72]. There appear to be two Blimp homologs in *Daphnia*. However, in one, the 5th finger is missing, and the third finger has two serines replacing the two cysteines of the finger. Consequently, the function of this finger (and the entire protein) may have changed substantially.

### C2H2 ZNF absent from one or more organisms

Zinc finger X-linked duplicated (ZXD) is a newly described C2H2 zinc finger family in bilaterians present in most chordates and has undergone duplication specifically in mammals. Among the bilaterians, humans and other mammals have 3 homologs for this family while nematodes, water fleas, sea urchins, chicken and frog all have one homolog including *Daphnia *(not shown). Interestingly, no homologs have been detected in insect lineage that has a sequenced genome and in *C. elegans*. Zinc finger X-linked duplicated family member C (ZXDC) along with its binding partner ZXDA, forms a complex that interacts with CIITA and regulates MHC II transcription [73,74]. The function of the other paralog ZXDB as well as the homolog in *C. elegans *is little understood.

CTCF and CTCFL (Figure 8) have 11 zinc fingers arranged in tandem. They act in part as "enhancer blockers" in vertebrates by binding to insulator elements. In flies, dCTCF binds to the Fab-8 insulator element between iab-7 and iab-8 [75]. Mammalian CTCF's are also involved in reading gene imprinting marks (i.e. Boris) at a high fraction of imprinted genes [76]. Recently, CTCF (along with YY1) has also been implicated in the global repression mechanism known as X-inactivation [77]. There are two human homologs, CTCF and CTCFL, but only one in *Daphnia *and *Drosophila*, and none in worms.

Yin Yang 1 (YY1) generally has 4 zinc-fingers. Recent phylogenetic analyses proposed that the YY1 gene has undergone independent duplication events in different lineages through retro-transposition. Two duplication events in placental mammals are believed to have given rise to the YY1, YY2, and REX1 (Reduced EXpression). A similar duplication event in flies produced the pleiohomeotic (Pho) and Pho-like genes [78]. The *Daphnia *Pho gene clusters with the *Drosophila *Pho (Figure 8). YY1 acts to activate or repress transcription in different contexts. In mammals, this gene appears to play multiple roles, including induction and patterning of the embryonic nervous system, differentiation within blood cell lineages, cell-cycle control, cell proliferation, differentiation, and apoptosis, DNA synthesis and packaging, and X-inactivation [79]. The fly homologs, Pho and phol, are classified as PcG (poly comb group) proteins that bind to PREs (PcG response elements that regulate homeotic genes). Pho and Phol act redundantly to repress homeotic gene expression in imaginal discs of the fly [80].

Hindsight (Hnt) in *Drosophila *is a homolog of Ras-Responsive Element Binding protein 1 (RREB1) in vertebrates. No homolog has been detected in worms, but *Daphnia *appears to have a single homolog (Additional file 1). The number of zinc fingers varies from species to species: 15 in humans, but only 10 in *Daphnia*. The *Pebbled (peb) *gene encodes the Hindsight protein, involved in morphogenetic processes and is expressed in several kinds of epithelial cells during development including extra embryonic amnioserosa, midgut, trachea, and the photoreceptor cells of the developing adult retina. In the amnioserosa, Hnt is required for embryonic germ band retraction and embryonic dorsal closure [81]. In tracheal development, it is required for the maintenance of epithelial integrity and assembly of apical extracellular structures known as taenidia [82]. During eye development, it is required for the accumulation of F actin in the apical tip of photoreceptor precursor cells in the ommatidial clusters, as well as in the developing rhabdomere during the pupal period [83]. HNT expression is also essential for maintaining epithelial integrity for amnioserosa, and retinal epithelium. Recently HNT has been shown to regulate Notch signaling in follicular epithelial development, which in turn alters cell differentiation and cell division. It is responsible for repressing String, Cut, and Hedgehog signaling, which are essential for regulating follicular cell proliferation [84]. The human homolog of HNT, RREB1 acts as a transcription factor that binds specifically to the RAS-responsive elements (RRE) of gene promoters. Recent investigations indicate that RREB1 is essential for spreading and migration of MCF-10A breast epithelial cells [85].

OAZ was apparently duplicated giving rise to two homologs in many vertebrates, including humans. OAZ is also called ZF423, while the closely related protein EHZF1 is called ZF521. No worm homolog has been detected, but a single homolog exists in *Daphnia *(Figure 5). Human and mouse homologs for this family have 30 zinc fingers, while *Drosophila *and *Daphnia *have fewer. OAZ/ZNF 423 and EHZF/ZNF521 are implicated in the control of olfactory epithelium, in B-lymphocyte differentiation, and in signal transduction by bone morphogenic protein (BMP). They are known to activate the BMP target genes *vent-2 *(*Xenopus*) and *ventx2 *(human) via interaction with SMAD [86]. ZNF521, in humans is known to regulate ontogenesis of the hemato-vascular system through BMP pathways. OAZ/ZNF423 can also repress BMPs by activating repressors of BMPs [87,88]. OAZ can apparently use different clusters of zinc fingers to interact with DNA, RNA or Protein [89]. In flies, 21 Zinc fingers are grouped into 4 clusters; the cluster near the amino terminus is assumed to bind DNA. DmOAZ is expressed throughout the life of flies and is strongest in posterior spiracles. Recent studies have shown that OAZ is involved in controlling posterior structure by regulating specific genes [90].

ZFAM9 is also known as Positive regulatory domain 13 (PRDM13) and is present in most vertebrates. A likely homolog of this gene is also present in *Drosophila *and *Daphnia *(Figure 8). However, the *C. elegans *genome had no homolog for this gene. All orthologs have 4 C2H2 zinc fingers. The function of these genes is unclear.

MTF (Metal-responsive Transcription Factor) have 6 tandem zinc-fingers. MTF activates metallothionein promoters in metazoans (Figure 3). MTF binds to the metal responsive element (MRE) and is involved in metal homeostasis and heavy-metal detoxification [91]. MTF in *Drosophila *appears to have a greater role in copper homeostasis than seen in vertebrates [92]. A single MTF homolog exists in *Daphnia *(not shown), but there appear to be no homologs in worms.

TFIIIA is a DNA-binding transcription factor that also binds RNA. It is generally required for 5sRNA gene expression in metazoans. TF3A's are poorly conserved between distantly related organisms. Vertebrate, insect, fungal, and plant sequences show within group similarity but only weak between group similarity [93,94]. TFIIIA usually has 9 zinc-fingers, as does the single homolog in *Daphnia *(not shown). In yeast, *S. pombe *has a 10th zinc finger following a long spacer, while *S. cereviseae *has a long spacer between the 8th and 9th fingers [95]. No homolog of TFIIIA has yet been identified in worms.

### C2H2 ZNF homologs of *Drosophila *developmental control genes

*Daphnia *have single homologs of the following well-known developmental control genes in *Drosophila*: Ovo, CI, LMD, OPA, SCRT, Slug, and ESG. In addition, *Daphnia *have three genes similar to the Odd gene family members Bowl, Bowel, and SOB (Figures S1 and S2). Although these genes encode C2H2 ZNF, other companion papers from the *Daphnia *consortium are intended to cover these in detail (personal communication).

## Discussion

Zinc-finger proteins probably represent the largest class of DNA-binding transcription factors in metazoan organisms, and as such, are likely to play critical roles in determining the extent to which various aspects of form and function are shared among taxa. The majority of these proteins are C2H2 zinc-finger proteins, many of which are already known to affect development and/or differentiation through a more or less direct effect on gene activation and/or repression.

A recent comparison of the full complement of C2H2 zinc-finger protein observed or predicted within worm, fly, and human genomes has lead to the tentative identification of nearly 40 orthologous groups shared between humans and invertebrates. From a phylogenetic perspective, the *Daphnia *genome would be expected to contain identifiable members for most of these groups. Using the reciprocal blast hit approach for estimating orthology, we uncovered 58 genes in *Daphnia *that appeared to be members of one of the 40 families conserved in bilaterians (Tables, 2, 3, 4 and 5). At least one member was identified for almost all families; only the JAZ family appeared to be absent from both *Daphnia *and *C. elegans*. All but two families had 3 or fewer members; only the SP and KLF families had more than three members each. Only the Odd-skipped and Snail families had 4 members each, while GLI, GFI, and Blimp had two members each. For all other families (33), only a single conserved member was identified in *Daphnia*. For 9 of these, a single conserved member was also present in each of the three other genomes; hence the latter genes appear to be relatively resistant to lineage specific deletion or expansion. Only three of the 40 families (including the most notable example, KLF) exhibited duplication or expansion in *Daphnia *relative to flies, but in many cases, gene duplications or expansions observed in flies and/or humans appeared to be absent in *Daphnia*. Thus the *Daphnia *genome appears to be relatively efficient with respect to the number of C2H2 ZNF homologs per family.

## Conclusions

Updating a previous analysis of C2H2 ZFP present in the common ancestor of bilaterians based on a survey of *Homo sapiens*, *Drosophila melanogaster *and *Caenorhabditis elegans*, we identified 58 well conserved C2H2 ZFP genes in *Daphnia *that belong to 40 distinct families. The *Daphnia *genome appears to be relatively efficient with respect to these well conserved C2H2 ZFP, since only 7 of the 40 gene families have more than one identified member. Worms have a comparable number of 6. In flies and humans, C2H2 ZFP gene expansions are more common, since these organisms display 15 and 24 multi-member families respectively. In contrast, only three of the well conserved C2H2 ZFP families have expanded in *Daphnia *relative to *Drosophila*, and in two of these cases, just one additional gene was found. The KLF/SP family in *Daphnia*, however, is significantly larger than that of *Drosophila*, and many of the additional members found in *Daphnia *appear to correspond to KLF 1/2/4 homologs, which are absent in *Drosophila*, but present in vertebrates.

## Methods

Identification of orthologs in *D. pulex*: Previously identified orthologs [2] that were present in the common ancestor of the bilaterians *Homo sapiens*, *Drosophila melanogaster*, and *Caenorhabditis elegans *were used as a focus for the present study. Protein sequences from each of the 3 different species belonging to 39 different classes of C2H2 zinc finger proteins were collected. Each of these sequences was used in turn as a query in a BLAST search against the v1.1 gene model annotations of the draft genome assembly of *D. pulex *to detect homologous protein sequences. High-scoring *Daphnia *sequences were examined to ensure a good overall match, and then used in a reciprocal BLAST against *Homo sapiens*, *Drosophila melanogaster *and *Caenorhabditis elegans*. Only those sequences that detected members of the same family represented by the original query sequence were retained as putative orthologs for those families. Other known C2H2 zinc finger binding genes were also used to search for any new families common to these bilaterians.

After the initial reciprocal BLAST approach, to search for additional gene members for the families, Hidden-Markov model (HMM) searches were conducted using the HMM profiles obtained from TreeFam for each of these 39 gene families. *Daphnia pulex *protein predictions containing all models were used to search with HMM profiles using HMMER 3.0 search. Only domains with an E-value < 0.1 were accepted, and any identified zinc finger gene was further manually inspected for their family characteristics. BLAST search was performed against the non redundant protein dataset at NCBI to confirm its family association. Only genes that appeared to be substantially complete and those that had approximately the same number of zinc fingers as other members of the proposed family were considered to be unambiguous members of that family. This approach identified additional homologs of Odd-skipped and Snail families and validated all the genes that were identified with reciprocal BLAST approach.

Alignments and phylogenetic analyses: *Daphnia pulex *homologs identified as above were combined with their respective family members from the other three bilaterians (*Homo sapiens*, *Drosophila melanogaster *and *Caenorhabditis elegans*; see Tables, 2, 3, 4 and 5) and used to create family-specific and/or multiple family alignments using the *Muscle *program (version 3.6) with 16 iterations and a standard Clustalw weighting scheme (gap opening extension, [96] closing and separation penalty of 10, 0.2, 4 and 1 respectively). The obtained alignment was then trimmed and converted to suitable format using trimAI (version 1.2)[97]. Phylogenetic trees were generated using Bayesian inference (*MrBayes*; version 3.1.2)[98] using WAG amino acid substitution matrix[99], empirically estimated amino acid frequencies plus gamma distribution of eight categories (WAG+F+Γ_8_). Successive runs were executed for a fixed number of generations with a sampling frequency of 100 and a burn-in parameter of 200. Runs were extended in each case until a convergence value of less than 0.03 was achieved. Since the multi-family trees each contained only an exclusive subset of the 40 total C2H2 zinc-finger families, internal branch patterns and statistics could be misleading with respect to the degree of relatedness between families. Hence, internal branches indicating a specific relationship between specific families within multi-family trees were collapsed into polytomies for presentation.

## Authors' contributions

GS established the overall concept and approach, and both YB and GS initiated gene identification and annotation. AS completed the bulk of the identification, annotation, organization, and documentation of genes, as well as producing all phylogenetic trees and writing early drafts of the manuscript. All authors read and approved the final manuscript.

## Supplementary Material

Additional file 1 Genes likely to be involved in oogenesis and/or pattern formation showing no expansion in *Drosophila *relative to *Daphnia*. Click here for file

Additional file 2 Genes likely to be involved in oogenesis and/or pattern formation showing expansions in *Drosophila *relative to *Daphnia*. Click here for file
