# Supplementary figures and images for: A survey of well conserved families of C2H2 zinc-finger genes in Daphnia
Source: BMC Genomics. 2010 Apr 30;11:276. doi: 10.1186/1471-2164-11-276 (PMC2889900; doi:10.1186/1471-2164-11-276)

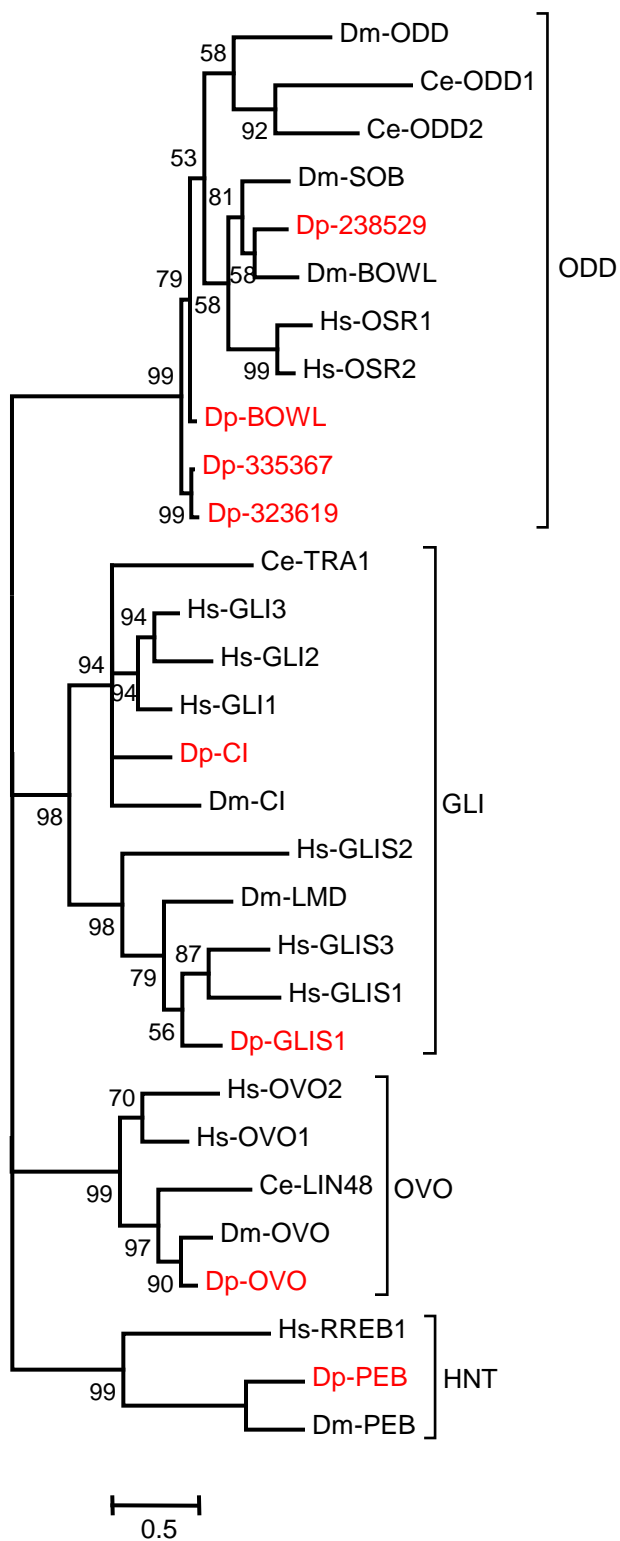

Supplement: Additional file 1 — Genes likely to be involved in oogenesis and/or pattern formation showing no expansion in Drosophila relative to Daphnia. [file 1471-2164-11-276-S1.PDF]

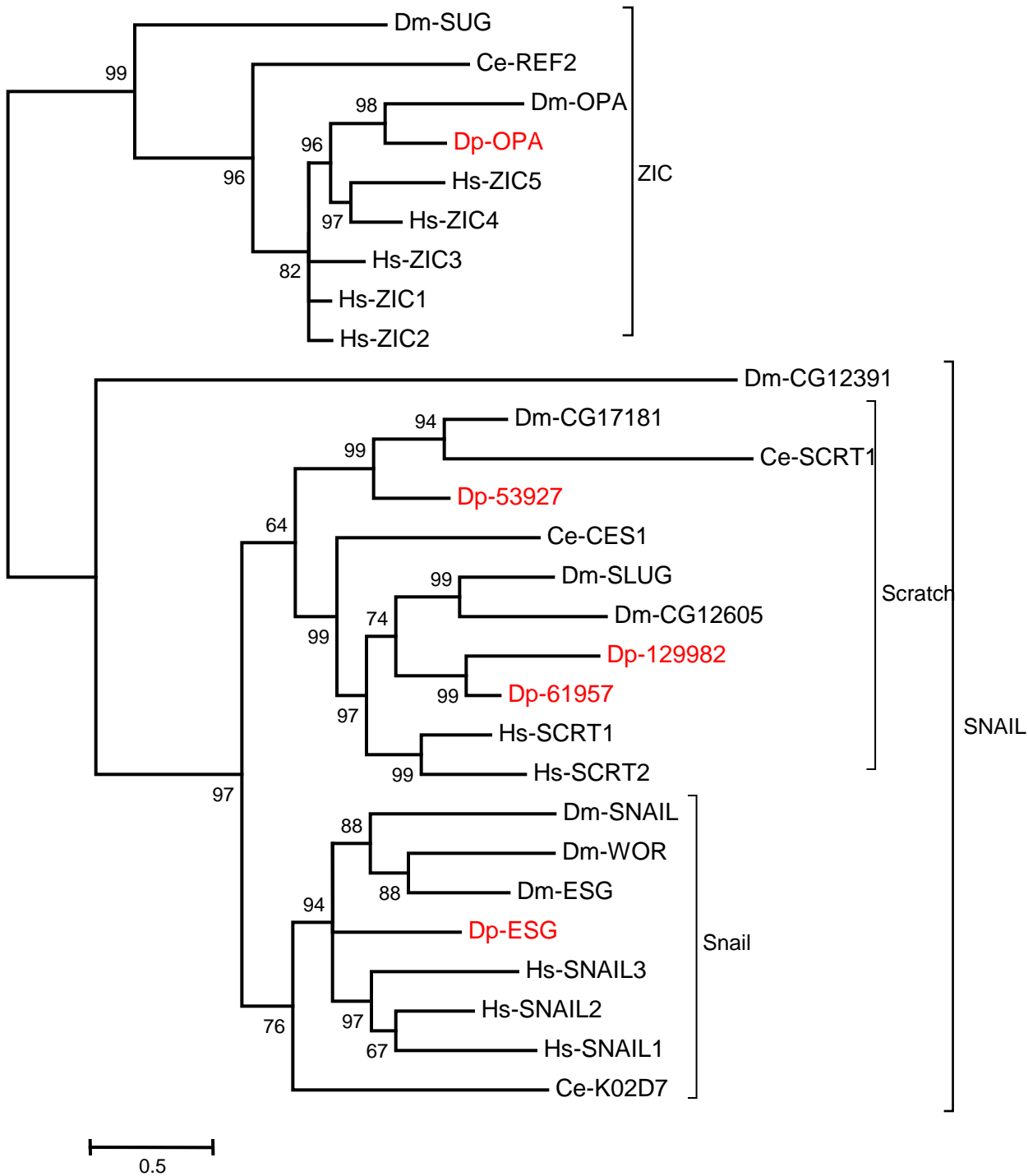

Supplement: Additional file 2 — Genes likely to be involved in oogenesis and/or pattern formation showing expansions in Drosophila relative to Daphnia. [file 1471-2164-11-276-S2.PDF]
